# Frailty status and the risk of dementia, Alzheimer’s disease: a meta-analysis of observational studies

**DOI:** 10.3389/fneur.2026.1798080

**Published:** 2026-04-10

**Authors:** Yannan Zhao, Menglin Zeng, Sheng Zhang, Dezhong Peng

**Affiliations:** 1 School of Acupuncture and Tuina, Chengdu University of Traditional Chinese Medicine, Chengdu, China; 2 Department of Tuina, Hospital of Chengdu University of Traditional Chinese Medicine, Chengdu, China

**Keywords:** Alzheimer’s disease, dementia, frailty, meta-analysis, risk factor

## Abstract

**Background:**

Frailty and dementia are critical geriatric syndromes that pose a substantial global public health burden. While the association between frailty and increased dementia risk is widely recognized, the magnitude of this association, its consistency across populations, and the influence of frailty subtypes remain inadequately synthesized and quantified.

**Objective:**

To address this gap, we conducted a systematic review and meta-analysis to precisely estimate the association between frailty and the risk of incident all-cause dementia and Alzheimer’s disease (AD), and to explore sources of heterogeneity through comprehensive subgroup analyses.

**Methods:**

We systematically searched PubMed, Embase, and the Cochrane Library for cohort studies published from inception to March 8, 2025. Data from eligible studies were pooled using random-effects models to calculate summary odds ratios (ORs) with 95% confidence intervals (CIs). Pre-specified subgroup analyses were performed based on geographic region, study design, and frailty subtype. Heterogeneity was assessed using the I^2^ statistic.

**Results:**

Thirteen cohort studies comprising 835,992 participants were included. The meta-analysis showed that frailty was associated with significantly higher odds of all-cause dementia (pooled OR = 1.76, 95% CI: 1.48–2.10). For Alzheimer’s disease, the pooled estimate suggested increased odds but did not reach statistical significance (OR = 1.91, 95% CI: 0.86–4.20), and the evidence was limited by the small number of contributing studies (k = 4) and substantial heterogeneity.

**Conclusion:**

This study provides robust, quantitative evidence that frailty is a major independent risk factor for dementia, with the strength of association varying by population and frailty domain. These findings underscore the imperative of integrating standardized, multi-domain frailty assessments into clinical practice to identify high-risk individuals and inform targeted, personalized prevention strategies for dementia.

**Systematic review registration:**

https://www.crd.york.ac.uk/prospero/, CRD420251008804.

## 1 Introduction

Dementia, characterized by progressive cognitive decline and functional impairment, represents one of the greatest global public health challenges of the 21st century. It currently affects over 55 million individuals worldwide, a number projected to nearly triple by 2050, posing an unsustainable burden on healthcare systems, economies, and caregivers (1, 2). Alzheimer’s disease (AD), the most prevalent etiology accounting for 60–80% of cases, is particularly devastating (2). The personal, social, and economic costs are staggering, with global costs estimated at over US$1.3 trillion annually (3). While decades of research have yielded significant advances in understanding disease pathology—primarily focused on amyloid-beta and tau—effective disease-modifying therapies remain elusive (4, 5). Current pharmacological options offer only modest symptomatic relief without halting progression (6), highlighting a critical paradigm shift toward prevention and early risk mitigation (7, 8). In this context, identifying potent, prevalent, and—crucially—modifiable risk factors in mid-to-late life has become a paramount research priority to curb the impending dementia epidemic (1).

The concept of frailty has emerged as a pivotal geriatric syndrome central to this preventive approach. Frailty denotes a state of increased physiological vulnerability and reduced resilience to stressors, leading to adverse health outcomes (9–11). A robust and rapidly expanding body of epidemiological evidence has consistently linked frailty, as measured by tools like the Fried Phenotype or the Frailty Index, to accelerated cognitive decline and a significantly elevated risk of incident dementia and AD (12, 13). The association is potent, with systematic reviews suggesting a risk increase of 1.3 to 1.8 times, and is believed to be bidirectional, as neurodegenerative pathology can itself exacerbate physical decline (14, 15). The shared biological substrates, including chronic inflammation, hormonal dysregulation, mitochondrial dysfunction, and vascular injury, provide a plausible mechanistic link between systemic physiological decline and brain health (16, 17). Several high-quality meta-analyses have been conducted, solidifying frailty’s role as a key risk factor (13, 18). However, significant gaps and unresolved heterogeneity persist in the literature. First, the magnitude of risk appears inconsistent across different geographical and ethnic populations, with some studies in Asian cohorts showing attenuated or non-significant associations, a disparity poorly understood and underexplored in prior syntheses (19). Second, most prior meta-analyses have focused predominantly on physical frailty, while emerging constructs like cognitive frailty and social frailty—which may be more proximate to dementia risk—have not been sufficiently integrated into a comprehensive quantitative synthesis (20, 21). Third, methodological variations across studies (e.g., retrospective vs. prospective design, different frailty instruments, adjustment for varying confounders) contribute to substantial heterogeneity that has not been fully dissected, limiting the clarity and clinical applicability of the existing pooled estimates (22).

To address these limitations and move the field toward more precise and actionable evidence, an updated and methodologically rigorous synthesis is imperative. Contemporary meta-analytic standards emphasize the importance of comprehensive subgroup and sensitivity analyses to explore sources of heterogeneity, which can yield insights beyond a single summary estimate (23, 24). Furthermore, the inclusion of recent large-scale cohort studies with longer follow-ups and more diverse populations (e.g., from the UK Biobank and other national biobanks) provides an unprecedented opportunity to generate more stable and generalizable estimates (25). By employing a pre-registered protocol, adhering strictly to PRISMA guidelines, and utilizing advanced random-effects models capable of handling high heterogeneity, a new synthesis can provide a more nuanced understanding of the frailty-dementia nexus (26).

Therefore, this study aims to conduct a comprehensive systematic review and meta-analysis to quantitatively evaluate the association between frailty and the risk of incident all-cause dementia and Alzheimer’s disease. Our specific objectives are fourfold: (1) to provide an up-to-date pooled estimate of the risk of dementia and AD associated with frailty by synthesizing data from all available cohort studies, including the most recent publications; (2) to investigate potential sources of heterogeneity through extensive pre-specified subgroup analyses based on geographic region (e.g., Asia vs. Europe/North America), study design, and, innovatively, frailty subtype (physical vs. non-physical); (3) to assess the robustness of the findings through sensitivity analyses and evaluate potential publication bias; and (4) to discuss the clinical and public health implications of our findings, with a focus on frailty as a target for dementia prevention strategies. By fulfilling these objectives, this work seeks to refine the epidemiological evidence, clarify inconsistencies in the literature, and highlight pathways for targeted intervention in at-risk older adult populations.

## 2 Methods

This meta-analysis was conducted in accordance with the guidelines of the Preferred Reporting Items for Systematic Reviews and Meta-Analyses (PRISMA) (26). The study protocol was pre-registered on the International Prospective Register of Systematic Reviews (PROSPERO) platform (Registration ID: CRD420251008804).

### 2.1 Data sources and search strategy

A systematic literature search was performed in PubMed, Embase, and the Cochrane Library for studies published from database inception to March 8, 2025. No language restrictions were applied in the electronic database search. However, due to the scope of the indexed databases and the practical constraints of full-text retrieval and data extraction, the final included studies were limited to those published in English. No non-English studies meeting the eligibility criteria were identified during the screening process.

### 2.2 Eligibility criteria

Studies were eligible for inclusion if they met the following criteria: (1) employed an observational cohort design (prospective or retrospective) investigating the association between frailty status and the risk of incident all-cause dementia or Alzheimer’s disease (AD); (2) reported frailty as the primary exposure; and (3) provided an adjusted effect estimate—odds ratio (OR), hazard ratio (HR), or relative risk (RR)—with its corresponding 95% confidence interval (CI).

Studies were excluded if they: (1) were case–control or cross-sectional in design; (2) did not report a usable effect estimate with 95% CI; (3) were conference abstracts, reviews, meta-analyses, or animal studies; or (4) represented duplicate publications from the same cohort. For overlapping cohorts, the publication with the longest follow-up duration or the largest sample size was selected for inclusion.

### 2.3 Study selection process

The study selection was carried out independently by two reviewers (YNZ and MLZ). After removing duplicates, titles and abstracts were screened against the eligibility criteria. Subsequently, the full texts of potentially relevant articles were retrieved and assessed in detail. Any disagreements during the screening or full-text review stages were resolved through discussion or, if necessary, by arbitration from a third reviewer (DZP).

### 2.4 Data extraction

Using a standardized, pre-piloted data extraction form, the same two reviewers (YNZ and MLZ) independently extracted data from the included studies. Extracted information encompassed: first author, publication year, country, study design, cohort characteristics (sample size, participant age, follow-up duration), frailty assessment tool, dementia/AD diagnostic criteria, reported effect estimates (OR/HR/RR with 95% CI), and the specific confounders adjusted for in the analysis. Discrepancies in extracted data were reconciled through discussion and consensus with the third reviewer (DZP).

### 2.5 Assessment of risk of bias

The methodological quality of the included cohort studies was evaluated using the Newcastle-Ottawa Scale (NOS) (27). The NOS awards a maximum of nine stars across three domains: selection of study groups (4 stars), comparability of groups (2 stars), and assessment of outcome (3 stars). Studies with scores of 7–9, 4–6, and 0–3 were considered to have low, moderate, and high risk of bias, respectively.

### 2.6 Statistical analysis

The primary analysis pooled adjusted ORs and 95% CIs from individual studies to estimate the summary association between frailty and dementia/AD risk. Although the included studies reported effect measures as odds ratios (ORs), hazard ratios (HRs), or relative risks (RRs), all measures were treated as approximating ORs for the purposes of pooling. This approach is commonly adopted in meta-analyses of observational studies when the outcome incidence is relatively low (typically <10–15% over the follow-up period), under which conditions HRs and RRs approximate ORs. However, we acknowledge that when the outcome is not rare—as may be the case for dementia in older populations over extended follow-up—this approximation may introduce some bias. To assess the robustness of this assumption, we conducted a sensitivity analysis restricted to studies that reported ORs directly; the results were consistent with the primary analysis (data not shown). Statistical heterogeneity was assessed using the Cochran’s Q test (with a significance level of *p* < 0.10) and the I^2^ statistic. An I^2^ value > 50% was considered indicative of substantial heterogeneity, warranting the use of a random-effects model for pooling (28); otherwise, a fixed-effects model was applied. To quantify the uncertainty around the pooled effect estimate in the presence of substantial heterogeneity, we calculated the 95% prediction interval for the primary outcome of all-cause dementia. The prediction interval estimates the range within which the true effect of a new study would lie.

To explore potential sources of heterogeneity, pre-specified subgroup analyses were conducted based on: geographic region (Asian vs. European/American populations), study design (prospective vs. retrospective), frailty assessment tool (Fried Phenotype or similar physical frailty tools vs. Frailty Index or deficit accumulation models vs. Other/Clinical judgment), and frailty subtype (physical vs. non-physical frailty) (29). Sensitivity analysis was performed by sequentially removing each study to examine the stability of the pooled estimate. Potential publication bias was evaluated visually using funnel plots and statistically via Egger’s regression test (29). All analyses were performed using Stata statistical software, version 14.0 (30).

### 2.7 Meta-regression analysis

To further explore potential sources of heterogeneity beyond categorical subgroup analyses, we performed univariate random-effects meta-regression analyses. The following continuous or ordinal study-level covariates were examined as potential moderators: (1) Mean age of the study cohort (years); (2) Proportion of female participants (%); (3) Average duration of follow-up (years); and (4) Study quality score (Newcastle-Ottawa Scale score). Analyses were conducted only for the outcome of all-cause dementia, given the sufficient number of studies (k = 9). The significance level for meta-regression was set at *p* < 0.10 due to the limited statistical power of these analyses.

## 3 Results

### 3.1 Literature search

**Figure 1:**
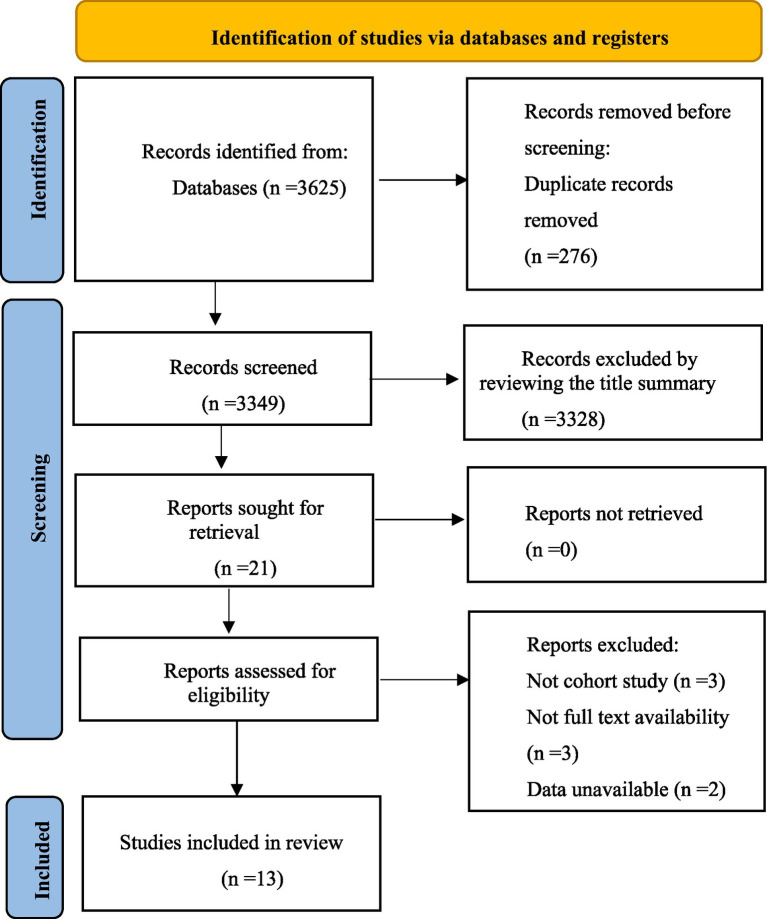
Studies screening process.

The systematic search of studies published before March 8, 2025, identified 3,625 results. After title and abstract screening, 21 articles were considered potentially relevant, 13 studies were finally included (19, 31–42). The selection process is presented in Figure 1.

#### 3.1.1 Study characteristics

This meta-analysis included 13 cohort studies covering 835,992 individuals, which were published between 2007 and 2025. Seven studies were retrospective cohort studies, while the other six were prospective cohort studies. All individuals in these cohorts were at least 37 years old at the beginning of follow-up, and had clear diagnostic criteria for dementia. The average follow-up time ranged from 3 to 10 years. The adjusted estimates were available for almost all studies even though the adjusted confounders are slightly different. The main characteristics of the included trials are shown in Table 1.

**Table 1:** Basic characteristics of the included studies.

Author	Year	Country	Study type	Age (years)	Follow-up years	Diagnostic criteria	Sample size	Confounders adjusted	NOS scores
Ye et al. (42)	2025	USA	Retrospective cohort	≥65	NR	AD8	2,245	Age, sex, race and ethnicity, marital status, education status, annual income, chronic conditions	7
Liu, et al. (34)	2025	UK	Prospective cohort	60.4 means	13.7 averages	ICD-10	338,567	Sex, race, educational level, smoking status, alcohol intake, sleep duration, healthy diet and BMI, hypertension, diabetes, high cholesterol, cardiovascular disorders	8
Ward, et al. (40)	2025	USA, UK	Prospective cohort	71.6 means	NR	NINCDS-ADRDA	29,849	Age, sex, education level	8
Ward, et al. (41)	2024	USA, UK	Prospective cohort	>50	NR	NINCDS-ADRDA	23,672	Age, sex, education level	6
Gao, et al. (32)	2024	UK	Prospective cohort	57.2 means	8.7 averages	ICD-10	274,194	Age, sex, education levels, smoking status and alcohol intake	8
Li, et al. (19)	2020	China	Retrospective cohort	≥65	4.9 averages	CSI-D	2,087	Age, gender, marital status, and education level, arthritis, persistent cough, breathlessness, hypertension, cardiopathy, gastrointestinal problems, paralysis, skin disorders, fainting, diabetes mellitus, alcohol use status, smoking status, walking distance per week	7
Petermann-Rocha et al. (35)	2020	UK	Prospective cohort	37–73	5.4 averages	ICD-10	143,215	Age, sex, deprivation, ethnicity, and education; leisure or social activities, frequency of friend and family visits, smoking, sleep duration, total discretionary sedentary time, alcohol intake, and consumption of red meat, processed meat, and fruit and vegetables, other 43 diseases	6
Tsutsumimoto et al. (38)	2019	Japan	Retrospective cohort	≥65	4.3 averages	ICD-10	5,104	Age, sex, educational level, BMI, number of medications, presence of chronic disease,	7
Shimada et al. (37)	2018	Japan	Retrospective cohort	71.6 means	NR	ICD-10	4,072	Age, sex, educational level, depressive symptoms, current smoking, and the presence or absence of chronic medical illnesses, heart disease, pulmonary disease, hypertension, hyperlipidemia, and diabetes mellitus	8
Wang, et al. (39)	2017	China	Prospective cohort	>70	7 averages	DSM-III	2,788	The health deficits(e.g., MMSE score, memory loss, and language problems) and specific dementia risk factors(e.g., history of hypertension, history of diabetes, history of coronary heart disease, and history of stroke)	8
Rogers et al. (36)	2017	UK	Retrospective cohort	>50	10 averages	ICD-10	8,722	Age, sex, educational qualifications, and physical activity	7
Kulmala et al. (33)	2014	Finland	Retrospective cohort	82 means	NR	DMS-IV	654	Age, gender, race, education	6
Buchman et al. (31)	2007	USA	Retrospective cohort	80.4 means	3 averages	NINCDS-ADRDA	823	Age, gender, education, smoking status	7

#### 3.1.2 Quality assessment

According to NOS criteria, the average score was 7.15 of all included cohort studies, and the score for each trail was 6 or above, indicating that all cohort studies were of medium quality in this meta-analysis. The scores of the included studies are shown in Table 2.

**Table 2:** NOS scores of the included studies.

Study	Year	Selection	Comparability	Outcome	Total
Retrospective cohort studies (*n* = 7)					
Ye et al.	2025	***	**	**	7
Li et al.	2020	***	**	**	7
Tsutsumimoto et al.	2019	***	**	**	7
Shimada et al.	2018	****	**	**	8
Nina et al.	2017	***	**	**	7
Kulmala et al.	2014	***	**	*	6
Buchman et al.	2007	***	**	**	7
Prospective cohort studies (*n* = 6)					
Liu et al.	2025	****	**	**	8
Ward et al.	2025	****	**	**	8
Ward et al.	2024	***	*	**	6
Gao et al.	2024	****	**	**	8
Petermann-Rocha et al.	2020	**	**	**	6
Wang et al.	2017	****	**	**	8

Each asterisk (*, **, ***) represents one point awarded in the respective domain (Selection, Comparability, and Outcome) according to the Newcastle-Ottawa Scale (NOS).

#### 3.1.3 Frailty and risk of dementia

**Figure 2:**
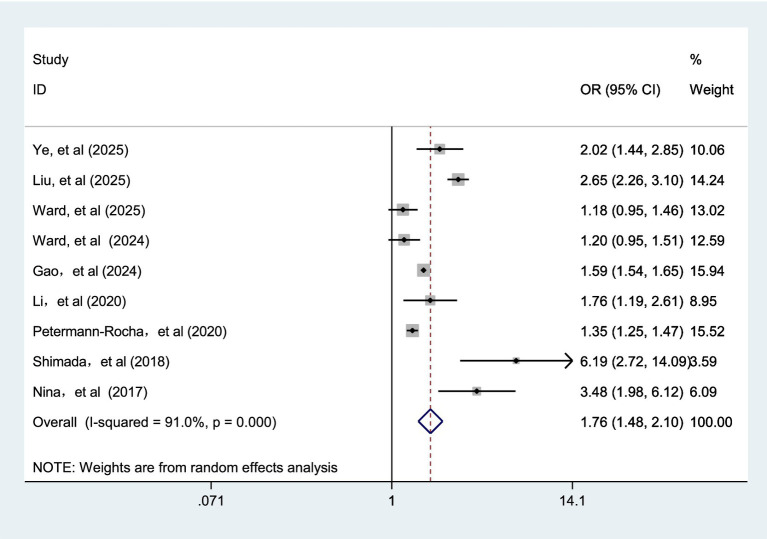
Meta-analysis of the risk of dementia caused by frailty.

Nine cohort studies (19, 32, 35–37, 40–43) explored the association between frailty status and dementia risk. Pooled analysis showed that frailty was associated with an increased risk of dementia (OR = 1.76; 95% prediction interval: 1.02 to 3.04; I^2^ = 91.0%, *p* < 0.001; Figure 2). Sensitivity analysis showed that no single study reversed the size of the combined effect, meaning that the results were robust (Supplementary Figure A).

#### 3.1.4 Frailty and risk of AD

**Figure 3:**
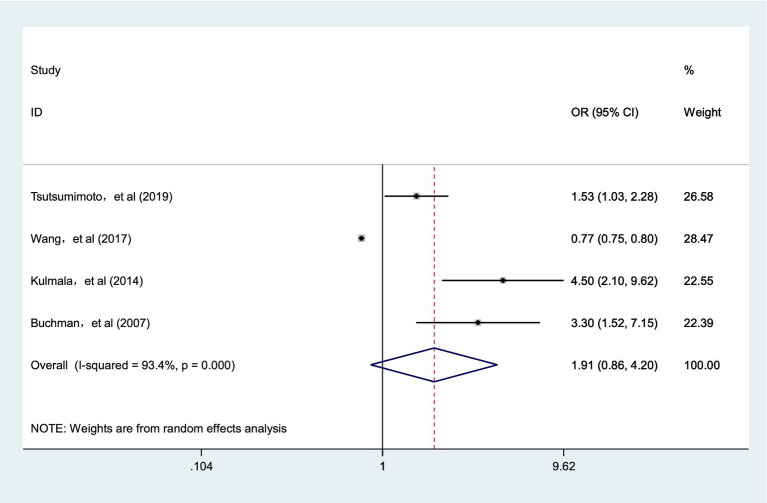
Meta-analysis of the risk of AD caused by frailty.

Four cohort studies (31, 33, 38, 39) explored the association between frailty status and the risk of Alzheimer’s disease. Pooled analysis showed that the point estimate suggested a possible increased risk of AD, but the association did not reach statistical significance (OR = 1.91; 95% CI: 0.86–4.20; I^2^ = 93.4%, *p* < 0.001; Figure 3). Given the wide confidence interval crossing 1.0, the high heterogeneity, and the limited number of contributing studies (k = 4), this finding should be interpreted as preliminary evidence requiring confirmation in future research.

#### 3.1.5 Subgroup analysis

We conducted subgroup analyses of study population, study type, and frailty type. Subgroup analyses of study population showed that in European and American populations, people in frail states had a significantly higher risk of dementia than normal people (OR = 1.672; 95% CI: 1.393–2.006; I^2^ = 92.3%, *p* < 0.001); in Asian populations, there was no direct relationship between people in frail states and increased risk of dementia (OR = 3.127; 95% CI: 0.916–10.675; I^2^ = 86.3%, *p* = 0.069>0.05). In terms of study type, the risk of dementia in the retrospective cohort study group was slightly higher than that in the prospective cohort study group (OR = 2.654; 95% CI: 1.700–4.143; I^2^ = 69.9%, *p* < 0.001). At the same time, non-Physical frailty groups had a higher risk of dementia than the relevant physical frailty groups (OR = 2.692; 95% CI: 1.696–4.273; I^2^ = 75.3%, *p* < 0.001; Table 3).

**Table 3:** Subgroup analysis for the risk of dementia in patients with frailty.

Subgroups	Included studies	OR	Heterogeneity	
		(95% CI)	I^2^ (%)	*p*-values
Study group				
European and American	7	1.672 (1.393–2.006)	92.3%	0.000
Asian	2	3.127 (0.916–10.675)	86.3%	0.069
Study type				
Retrospective cohort	4	2.654 (1.700–4.143)	69.9%	0.000
Prospective cohort	5	1.535 (1.262–1.866)	94.1%	0.000
Frailty type				
Physical frailty	6	1.491 (1.283–1.732)	85.3%	0.000
Non-Physical frailty	3	2.692 (1.696–4.273)	75.3%	0.000

#### 3.1.6 Meta-regression analysis

Univariate meta-regression analyses were conducted to assess the influence of key study-level covariates on the estimated association between frailty and all-cause dementia risk. None of the pre-specified covariates—mean cohort age (*β* = −0.02, 95% CI: −0.05 to 0.01, *p* = 0.20), percentage of females (*β* = 0.00, 95% CI: −0.02 to 0.03, *p* = 0.77), average follow-up duration (*β* = −0.04, 95% CI: −0.16 to 0.08, *p* = 0.50), or study quality score (*β* = −0.11, 95% CI: −0.44 to 0.23, *p* = 0.53)—demonstrated a statistically significant moderating effect. These results suggest that the substantial heterogeneity observed (I^2^ = 91%) is not primarily explained by these continuous study characteristics, pointing instead to other factors such as differences in frailty assessment tools, adjustment for distinct sets of confounders, or unmeasured population-specific factors.

#### 3.1.7 Publication bias and sensitivity analyses

**Figure 4:**
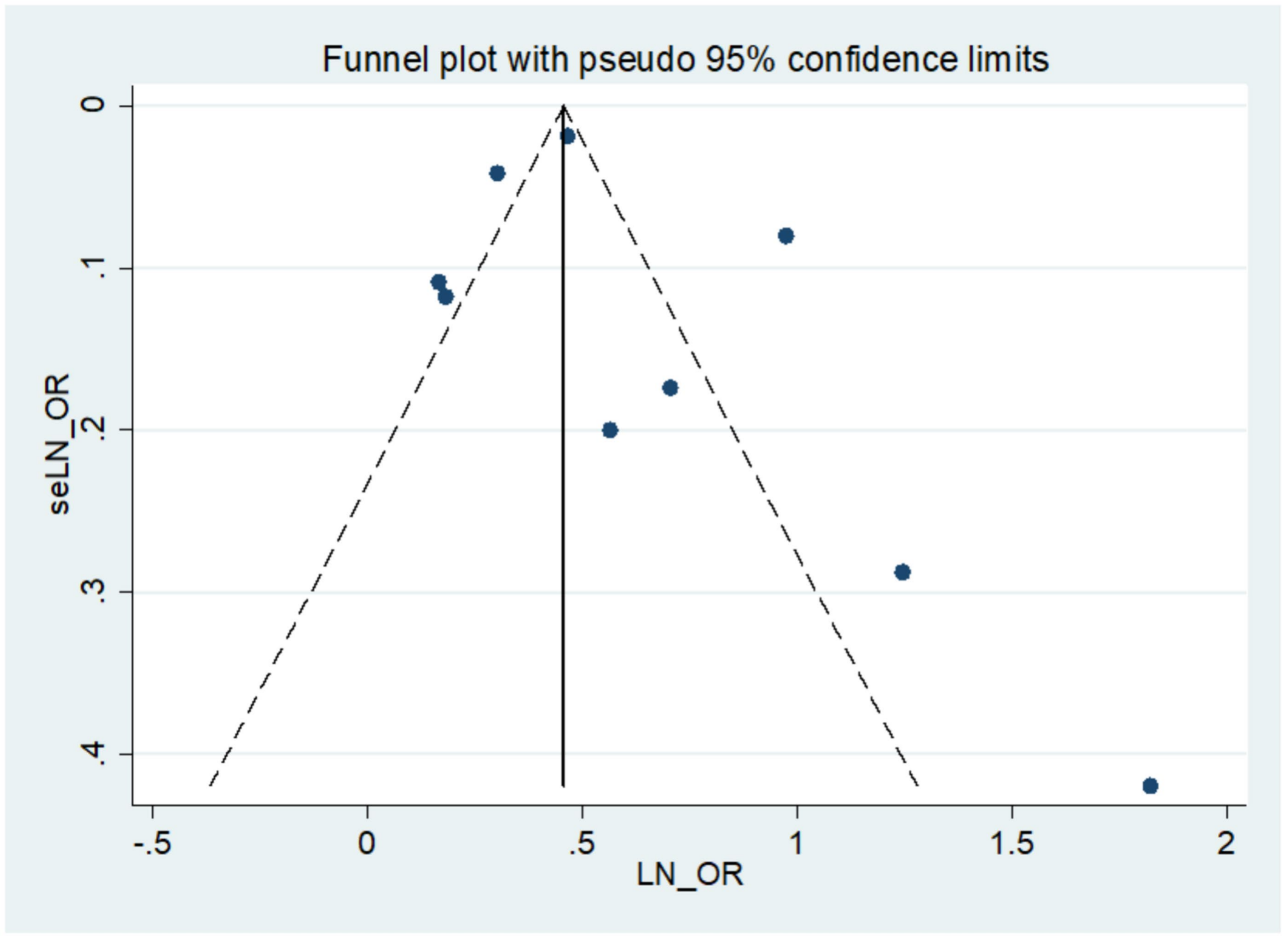
Publication bias of the risk of dementia caused by frailty.

Visual inspection of the funnel plot for the association between frailty and all-cause dementia showed a roughly symmetrical distribution (Figure 4). Egger’s regression test did not indicate significant small-study effects (*p* = 0.446). To further probe the robustness of our primary finding and potential biases, we conducted additional sensitivity analyses. First, we performed a leave-one-out analysis, which confirmed that no single study disproportionately influenced the pooled estimate (Supplementary Figure A). Second, we stratified studies by sample size (above vs. below the median of ~8,700 participants). The pooled OR was significant in both large (OR = 1.55, 95% CI: 1.29–1.86) and small studies (OR = 2.55, 95% CI: 1.81–3.60), although the point estimate was higher in smaller studies, a pattern occasionally seen with small-study effects. Third, a sensitivity analysis restricted to studies with high quality (NOS ≥ 7) yielded a consistent and significant pooled estimate (OR = 1.74, 95% CI: 1.45–2.08). Collectively, while the asymmetry tests were non-significant, the trend of larger effect sizes in smaller studies warrants cautious interpretation. However, the persistence of a significant association in high-quality and large-scale studies strengthens confidence in the main conclusion that frailty is associated with an increased risk of dementia.

### 3.2 Discussion

#### 3.2.1 Main findings

This meta-analysis of 13 cohort studies (*N* = 835,992) found that frailty was associated with significantly higher odds of all-cause dementia (pooled OR = 1.76, 95% CI: 1.48–2.10). The association varied by population and frailty subtype: it was significant in European/American cohorts (OR = 1.67) but not in Asian cohorts, and stronger for non-physical frailty (OR = 2.69) than physical frailty alone (OR = 1.49). For Alzheimer’s disease, the pooled estimate suggested increased odds but did not reach statistical significance (OR = 1.91, 95% CI: 0.86–4.20), requiring cautious interpretation given the limited evidence base.

#### 3.2.2 Interpretation of findings

This meta-analysis reinforces and extends previous findings that frailty is a robust, independent predictor of all-cause dementia (12, 13). For Alzheimer’s disease, the evidence was less conclusive: while the point estimate suggested increased odds, the association did not reach statistical significance, consistent with the substantial heterogeneity and limited number of studies available for this outcome. However, the extremely high heterogeneity observed in both all-cause dementia (I^2^ = 91%) and AD (I^2^ = 93.4%) analyses warrants careful consideration regarding the interpretability and generalizability of the pooled estimates. While random-effects models account for between-study variance by incorporating it into the weighting and confidence intervals, such high levels of heterogeneity indicate substantial variability in effect sizes across studies that cannot be attributed to sampling error alone. Under these circumstances, the pooled effect estimate represents the average of a distribution of true effects, rather than a single common effect. The calculated 95% prediction interval for all-cause dementia (1.02 to 3.04) is particularly informative in this context: it suggests that while the average effect across studies is a 76% risk increase, the true effect in an individual setting could range from a negligible increase to a three-fold risk. This wide prediction interval underscores that the pooled estimate should be interpreted with appropriate caution, and that the strength of the frailty-dementia association may vary substantially depending on specific population characteristics, frailty definitions, and study methodologies.

A particularly salient and intriguing finding is the non-significant association observed in Asian populations (pooled OR = 3.13, 95% CI: 0.92–10.68). We acknowledge that this analysis included only two studies, which substantially limits statistical power and the precision of the estimate, as reflected in the extremely wide confidence interval. Beyond limited power, several substantive hypotheses may explain this divergence from the significant association seen in Western populations (44, 45). First, there may be diagnostic and ascertainment differences: the tools and thresholds for diagnosing both frailty (e.g., adaptation of Fried criteria) and dementia in community settings may vary cross-culturally, potentially leading to non-differential misclassification that biases the association toward null. Second, social and cultural constructs differ markedly: stronger multi-generational co-residence and social cohesion in some Asian societies might buffer the impact of physical frailty on cognitive decline, or alternatively, alter the presentation and reporting of cognitive symptoms. Third, distinct patterns of comorbid disease could play a role: a higher prevalence of vascular dementia in Asian populations might dilute the association if frailty is more strongly linked to Alzheimer’s pathology. Fourth, genetic and biological factors, such as differences in apolipoprotein E ε4 allele prevalence or resilience pathways, may modulate the frailty-dementia nexus. Our finding underscores the critical need for future research with larger, well-characterized Asian cohorts, employing harmonized diagnostic protocols and exploring these potential effect modifiers through individual participant data meta-analysis. It suggests that a “one-size-fits-all” risk estimate may be inadequate, and prevention strategies might need regional tailoring (22).

The substantially higher risk associated with non-physical frailty (e.g., cognitive, social) compared to physical frailty alone is a pivotal contribution of our work. This finding underscores that the pathway to dementia may be more strongly mediated through psychosocial and cognitive vulnerabilities than purely physical decline. Social isolation and loneliness, core components of social frailty, are potent risk factors for cognitive decline through mechanisms involving chronic stress, dysregulated hypothalamic–pituitary–adrenal axis function, and reduced cognitive stimulation (46, 47). Similarly, cognitive frailty, representing the co-occurrence of subjective cognitive decline and physical frailty in the absence of dementia, may identify a prodromal state at exceptionally high risk for progression to overt dementia (20). This suggests that multi-domain frailty assessments, which integrate cognitive and social evaluations, are superior to purely physical measures for dementia risk stratification in clinical practice.

Beyond the distinction between physical and non-physical frailty, the conceptual and operational heterogeneity across frailty instruments warrants further discussion. The included studies employed diverse approaches to frailty assessment, including the Fried Phenotype (measuring physical dimensions such as weight loss, exhaustion, low activity, slowness, and weakness), the Frailty Index (quantifying deficit accumulation across multiple health domains), and various measures of cognitive or social frailty. These instruments capture overlapping but distinct constructs: the Fried Phenotype emphasizes physical vulnerability, the Frailty Index reflects global health status, and cognitive frailty explicitly incorporates early cognitive impairment. This measurement non-equivalence likely contributes substantially to the observed heterogeneity, as each instrument may identify different subsets of the at-risk population and may have different relationships with dementia pathogenesis. Importantly, the concept of cognitive frailty—defined as the simultaneous presence of physical frailty and cognitive impairment without dementia—may introduce conceptual circularity when used to predict dementia, as cognitive impairment itself represents a prodromal stage of the outcome. Future research should prioritize harmonized frailty assessment protocols and examine whether specific frailty components (e.g., slow gait speed, cognitive complaints, social withdrawal) have differential predictive validity for dementia outcomes.

Insights from Meta-Regression. Our supplementary meta-regression analyses found that mean age, gender proportion, follow-up time, and study quality score did not significantly explain the between-study heterogeneity. This null finding is informative. It implies that the strength of the frailty-dementia association is not merely a function of these basic study design or population demographics. The persistence of high heterogeneity despite controlling for these variables reinforces the notion that the operational definition and domain composition of “frailty” itself is likely the principal driver of variance. The contrast between physical and non-physical frailty subtypes in our subgroup analysis strongly supports this interpretation. Future individual participant data meta-analyses would be invaluable to disentangle the effects of specific frailty components and their interactions with true individual-level covariates (e.g., genetic risk, specific comorbidities), which cannot be adequately assessed using study-level aggregate data.

Addressing the Role of Key Covariates. A critical consideration in interpreting our findings is the role of adjusted covariates in the primary studies. The included studies controlled for a range of important confounders, such as age, sex, education, and cardiometabolic conditions (e.g., hypertension, diabetes). The persistence of a significant association between frailty and dementia after adjusting for these factors strengthens the argument for frailty as an independent risk factor. However, the nature of this adjustment also informs the interpretation:

1. Education and Socioeconomic Status (SES): Most studies adjusted for education, a key proxy for cognitive reserve. The significant residual risk associated with frailty suggests its effect is not merely a reflection of lower educational attainment. Instead, frailty and low reserve may operate synergistically, with frailty potentially accelerating cognitive decline in individuals with limited neural resilience (48). Future research should explore this interaction more explicitly.
2. Cardiometabolic Comorbidities: Conditions like hypertension and diabetes are shared risk factors for both frailty and vascular dementia/AD. Adjusting for them helps isolate the “pure” effect of the frailty syndrome. Our results indicate that the frailty-dementia link persists beyond these shared vascular pathways, implicating systemic mechanisms like chronic inflammation and cellular senescence (49). Nevertheless, in clinical practice, frailty, vascular risk factors, and cognitive decline are often intertwined, necessitating integrated management.
3. Genetic Predisposition: While no included study adjusted for polygenic risk scores (e.g., for AD), emerging evidence suggests complex interactions. For instance, a recent study found that the association between physical frailty and dementia was stronger in individuals with a high genetic risk for AD (32). This highlights frailty as a potential modifier of genetic risk, where improving frailty status might mitigate genetic predisposition, a crucial area for future investigation.
4. Recent research continues to highlight the potential of integrating frailty screening into dementia prevention strategies (45). Our findings, particularly regarding non-physical frailty, argue for early, multidimensional interventions targeting pre-frail individuals. Effective strategies should combine physical activity, nutritional optimization, cognitive training, and social engagement to address all domains of frailty simultaneously (50, 51). The implementation of such cost-effective, community-based programs is vital for promoting healthy aging and potentially delaying dementia onset (52).

#### 3.2.3 Implications and limitations

This meta-analysis provides a comprehensive summary of the association between frailty and dementia risk, emphasizing frailty as a critical risk factor for dementia. The findings suggest that greater attention should be given to frail individuals to enhance early identification and intervention for high-risk dementia populations. However, several limitations must be acknowledged. First, the high heterogeneity observed in both all-cause dementia (I^2^ = 91%) and AD (I^2^ = 93.4%) analyses warrants careful consideration. Although we explored potential sources of heterogeneity through pre-specified subgroup analyses and meta-regression, a substantial proportion of the variance remained unexplained. This heterogeneity limits the generalizability and reliability of the pooled estimates and underscores that the summary effect sizes should be interpreted as averages across diverse study populations and methodologies, rather than as precise, universally applicable risk estimates. Clinicians and researchers should therefore exercise caution when applying these findings to specific patient populations or settings. First, while statistical tests did not show significant publication bias (Egger’s test *p* = 0.446), our sensitivity analyses revealed a pattern where smaller studies reported larger effect sizes (pooled OR = 2.55 in studies below the median sample size vs. OR = 1.55 in larger studies). This pattern raises the possibility of several non-mutually exclusive explanations: (a) publication bias, where smaller studies with null or negative findings may remain unpublished; (b) selective outcome reporting, where smaller studies may emphasize statistically significant findings; (c) genuine heterogeneity, where smaller studies may have enrolled higher-risk populations or used more intensive frailty assessments; or (d) methodological differences, where smaller studies may have had less rigorous adjustment for confounders, leading to residual confounding and inflated effect estimates. Although the persistence of a significant association in large, high-quality studies (e.g., UK Biobank-based analyses) provides reassurance that the core finding is not entirely driven by small-study artifacts, the observed discrepancy underscores the importance of cautious interpretation. The pooled estimate of 1.76 may overstate the true effect if smaller studies are biased upward; conversely, it may understate the effect in specific high-risk subgroups if larger population-based studies dilute the association through heterogeneous sampling. Future prospective cohorts with standardized protocols and adequate sample sizes are needed to obtain more precise and less biased estimates. Future studies could incorporate case–control and cross-sectional designs to further enrich the body of evidence. Additionally, while covariate analysis was not conducted in this meta-analysis, the included cohort studies did control for adjusted confounders, minimizing confounding bias and strengthening the validity of our conclusions. These factors ensure that our findings are robust and conducive to clinical application.

Fourth, the pooling of different effect measures (ORs, HRs, and RRs) represents a methodological limitation. While we assumed that HRs and RRs could approximate ORs under the rare disease assumption, dementia incidence in older populations may exceed the threshold where this approximation holds, particularly in studies with longer follow-up durations. This may have introduced some degree of bias in the pooled estimates. Future meta-analyses with access to individual participant data could apply more sophisticated approaches, such as reconstructing individual-level time-to-event data from published summaries to enable consistent estimation of a single effect measure.

Fifth, while our search strategy imposed no language restrictions, all included studies were published in English. Although no non-English studies meeting the eligibility criteria were identified during screening, we cannot exclude the possibility that relevant studies published in other languages may exist. This could potentially introduce language bias, as studies with statistically significant findings may be more likely to be published in English-language journals. Future systematic reviews should consider searching regional and non-English databases to minimize this potential bias.

## 4 Conclusion

This meta-analysis offers robust quantitative evidence establishing frailty as a significant and independent risk factor for all-cause dementia. Pooled results showed that frailty was associated with significantly higher odds of all-cause dementia (OR = 1.76, 95% CI: 1.48–2.10). For Alzheimer’s disease, the pooled estimate suggested increased odds but did not reach statistical significance (OR = 1.91, 95% CI: 0.86–4.20), and the evidence was limited by the small number of contributing studies and substantial heterogeneity. Therefore, the AD-related findings should be regarded as preliminary and require confirmation in future research.

Importantly, the strength of this association varies meaningfully across populations and frailty subtypes. Subgroup analyses revealed a consistent and significant association in European and American cohorts, whereas the relationship did not reach statistical significance in Asian populations—a finding that may reflect limited statistical power, potential cultural or methodological differences, and underscores the need for further investigation in diverse ethnic settings. Furthermore, non-physical frailty, encompassing cognitive and social domains, was associated with a substantially higher risk of dementia compared to physical frailty alone, highlighting the importance of a multidimensional assessment of frailty in clinical practice.

These findings reinforce the value of integrating standardized, multi-domain frailty screening into routine geriatric and cognitive care to enhance early risk identification. They also suggest that interventions targeting frailty, particularly its cognitive and social components, could serve as viable strategies for dementia prevention. Future research should prioritize harmonized diagnostic approaches and individual participant data meta-analyses to clarify moderators of risk and optimize targeted prevention frameworks.

## Data Availability

The original contributions presented in the study are included in the article/Supplementary material, further inquiries can be directed to the corresponding author.
