# Supplementary material for: Frailty status and the risk of dementia, Alzheimer’s disease: a meta-analysis of observational studies
Source: Front Neurol. 2026 Apr 10;17:1798080. doi: 10.3389/fneur.2026.1798080 (PMC13106089; doi:10.3389/fneur.2026.1798080)
Supplement: Supplementary file 1 [file Table_1.DOCX]

Supplementary Material

# Supplementary Figures and Tables

## Supplementary Figures


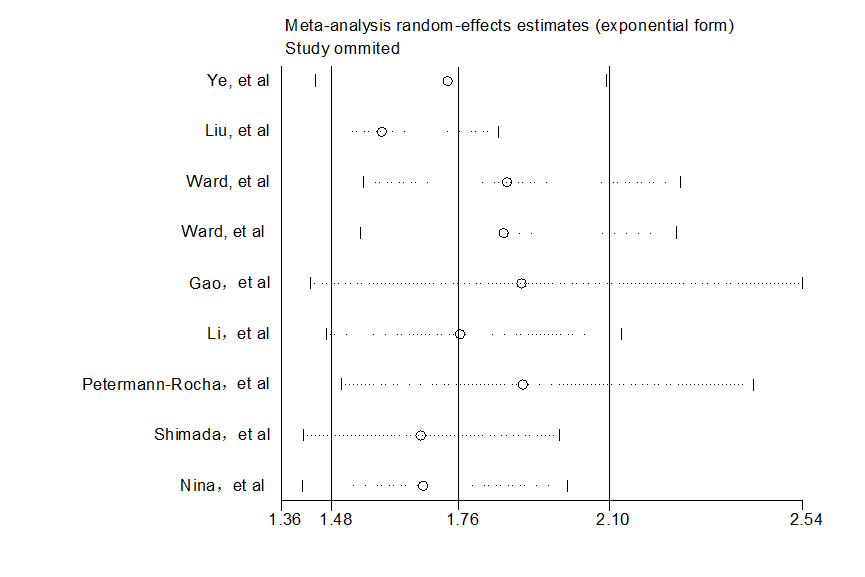


**Figure A**: Sensitivity analysis of the risk of dementia caused by frailty

## Supplementary Tables

**Table 1** The full search strategy of PubMed (2025.03.08)

| No. | Query | Results |
| --- | --- | --- |
| 1 | "Frailty"[Mesh] | 12,685 |
| 2 | ((Frailt*[Title/Abstract]) OR (Frailness[Title/Abstract])) OR (Debilit*[Title/Abstract]) | 71,138 |
| 3 | ("Frailty"[Mesh]) OR (((Frailt*[Title/Abstract]) OR (Frailness[Title/Abstract])) OR (Debilit*[Title/Abstract])) | 71,759 |
| 4 | "Alzheimer Disease"[Mesh] | 131,700 |
| 5 | "Dementia"[Mesh] | 224,584 |
| 6 | ("Alzheimer Disease"[Mesh]) OR ("Dementia"[Mesh]) | 224,584 |
| 7 | (((Alzheimer Type Dementia[Title/Abstract]) OR (Alzheimer* Diseases[Title/Abstract])) OR (Alzheimer Dementia*[Title/Abstract])) OR (Senile Dementia*[Title/Abstract]) | 5,986 |
| 8 | (Dementia*[Title/Abstract]) OR (Amentia*[Title/Abstract]) | 162,002 |
| 9 | ((((Alzheimer Type Dementia[Title/Abstract]) OR (Alzheimer* Diseases[Title/Abstract])) OR (Alzheimer Dementia*[Title/Abstract])) OR (Senile Dementia*[Title/Abstract])) OR ((Dementia*[Title/Abstract]) OR (Amentia*[Title/Abstract])) | 163,175 |
| 10 | (("Alzheimer Disease"[Mesh]) OR ("Dementia"[Mesh])) OR (((((Alzheimer Type Dementia[Title/Abstract]) OR (Alzheimer* Diseases[Title/Abstract])) OR (Alzheimer Dementia*[Title/Abstract])) OR (Senile Dementia*[Title/Abstract])) OR ((Dementia*[Title/Abstract]) OR (Amentia*[Title/Abstract]))) | 288,865 |
| 11 | "Risk"[Mesh] | 1,462,002 |
| 12 | risk*[Title/Abstract] | 3,303,427 |
| 13 | ("Risk"[Mesh]) OR (risk*[Title/Abstract]) | 3,807,031 |
| 14 | ((("Frailty"[Mesh]) OR (((Frailt*[Title/Abstract]) OR (Frailness[Title/Abstract])) OR (Debilit*[Title/Abstract]))) AND ((("Alzheimer Disease"[Mesh]) OR ("Dementia"[Mesh])) OR (((((Alzheimer Type Dementia[Title/Abstract]) OR (Alzheimer* Diseases[Title/Abstract])) OR (Alzheimer Dementia*[Title/Abstract])) OR (Senile Dementia*[Title/Abstract])) OR ((Dementia*[Title/Abstract]) OR (Amentia*[Title/Abstract]))))) AND (("Risk"[Mesh]) OR (risk*[Title/Abstract])) | 1,242 |

**Table 2** The full search strategy of Embase (2025.03.08)

| No. | Query | Results |
| --- | --- | --- |
| 1 | 'frailty'/exp | 35,934 |
| 2 | 'frailt*':ab,ti OR 'frailness':ab,ti | 46,518 |
| 3 | #1 OR #2 | 52,244 |
| 4 | 'alzheimer disease'/exp | 274,877 |
| 5 | 'alzheimer type dementia':ab,ti OR 'alzheimer* diseases':ab,ti OR 'alzheimer dementia*':ab,ti OR 'senile dementia*':ab,ti | 6,269 |
| 6 | #4 OR #5 | 277,175 |
| 7 | 'dementia'/exp | 497,020 |
| 8 | 'dementia*':ab,ti OR 'amentia*':ab,ti | 220,318 |
| 9 | #7 OR #8 | 531,188 |
| 10 | #6 OR #9 | 531,258 |
| 11 | risk | 5,531,116 |
| 12 | 'risk*':ab,ti | 4,702,213 |
| 13 | #11 OR #12 | 5,709,725 |
| 14 | #3 AND #10 AND #13 | 2,254 |

**Table 3** The full search strategy of Cochrane library (2025.03.08)

| No. | Query | Results |
| --- | --- | --- |
| 1 | MeSH descriptor: [Frailty] explode all trees | 878 |
| 2 | (Frailt*):ab,ti,kw OR (Frailness):ab,ti,kw | 4,157 |
| 3 | #1 OR #2 | 4,157 |
| 4 | MeSH descriptor: [Alzheimer Disease] explode all trees | 5,516 |
| 5 | (Alzheimer Type Dementia ):ab,ti,kw OR (Alzheimer* Diseases):ab,ti,kw OR (Alzheimer Dementia*):ab,ti,kw OR (Senile Dementia*):ab,ti,kw | 7,510 |
| 6 | #4 OR #5 | 10,411 |
| 7 | MeSH descriptor: [Dementia] explode all trees | 9,755 |
| 8 | (Dementia*):ab,ti,kw OR (Amentia*):ab,ti,kw | 18,591 |
| 9 | #7 OR #8 | 21,991 |
| 10 | #6 OR #9 | 22,430 |
| 11 | MeSH descriptor: [Risk] explode all trees | 55,436 |
| 12 | ( risk*):ab,ti,kw | 325,303 |
| 13 | #11 OR #12 | 329,113 |
| 14 | #3 AND #10 AND #13 | 129 |
